# Hughlings Jackson

**Published:** 1915-11-27

**Authors:** 


### HUGHLINGS JACKSON.

So rapid is the flight of time in these crowded days that it is hardly ungenerous to suppose that not a few, at least of the junior members of the profession, would confess ignorance of the special reasons for placing the name of Hughlings Jackson in a position of supreme honour. Apart from the pressure of modern work and the claim of this to attention, there are at least three circumstances which explain, if they do not excuse, such ignorance. First the man himself had in a very large measure the retiring modesty which lends a charm to true greatness; he had no ability for self-assertion, and the full measure of his achievement was hardly known save to those who enjoyed his intimacy and confidence. Secondly, his scattered writings had so wide a range and were pitched on so high a level, that only the prepared mind could fully appreciate them; time and effort and training are necessary to follow many of his flights and to learn the importance of his carefully qualified propositions. A third reason is the secure and unchallenged character of his conclusions; these have penetrated so intimately into the accepted positions of to-day, that it is difficult to realise how recent is their birth. Further, it may be admitted that we are yet too near the time of Hughlings Jackson to see his work in its due proportion and proper perspective. History will, of course, adjust these, and in the meantime we are in the period when the active work of a great man has ceased, and the impartial verdict of time is not yet pronounced. Towards that verdict a contemporary judgment has some duty, and we therefore welcome two recent efforts to place before the new generations the nature of the work which Hughlings Jackson accomplished.

By a happy inspiration the editors of *Brain* have been prompted to issue a special number of their journal containing a careful exposition of Hughlings Jackson's teaching on aphasia and a reprint of many of his original papers. As this work has been undertaken by Dr. Head it is superfluous to say that it has been accomplished with all thoroughness, good judgment, and sympathy; it is a worthy discharge of a debt which the present owes alike to the past and to the future. An equal tribute must be paid to the contribution made by Dr. James Taylor in his presidential address to the Neurological Section of the Royal Society of Medicine, and entitled "The Ophthalmological Observations of Hughlings Jackson." Dr. Taylor speaks with fullness of knowledge both of the man and of his work, and he will recognise no mean compliment in the remark that he has successfully followed and applied the teachings of his master and friend. The use of the ophthalmoscope and the evaluation of visual disturbances in the diagnosis and identification of nervous and other lesions is now a familiar theme; how much Hughlings Jackson contributed to this end is ably presented in Dr. Taylor's address. Larger and wider and more complicated conclusions are associated with Hughlings Jackson's name, but they all rest on patience and accuracy in observation, and on close reasoning united to insight and a scientific use of the imagination. The two contributions which we here note are the more important seeing that they supply materials out of which medical history will in due time be constructed.

